# How did you know you got the right pill? Prescription opioid identification and measurement error in the abuse deterrent formulation era

**DOI:** 10.1186/1940-0640-10-S1-A16

**Published:** 2015-02-20

**Authors:** Traci C Green, Carolyn Griffel, Taryn Dailey, Priyanka Garg, Eileen Thorley, Courtney Kaczmarsky, Theresa Cassidy, Stephen F Butler

**Affiliations:** 1 Inflexxion, Inc., Newton, MA, 02155, USA

## Background

Self-report of nonmedical prescription opioid use (NMPU) is a cornerstone of drug abuse surveillance, policymaking, and treatment service planning, but misclassification creates bias and may confuse or undermine NMPU estimates [1]. We detected old OxyContin (OC) abuse reports long after a reformulated version (OP) was released (August 2010). This study explored sources of possible NMPU misclassification and proposed solutions.

## Materials and methods

A mixed-methods approach identified demographic, behavioral, and cognitive factors influencing endorsement of old formulations in: a) multivariable regression analyses of NMPU data from the ASI-MV surveillance program [2] examined predictors of endorsing old (vs. new) formulations during the post-reformulation period (n = 8032); b) prevalence estimates of OC availability from an online recreational drug user forum survey (fall 2013; n = 459); and c) semistructured interviews (n = 29) and cognitive interviews (n = 7) among residential and outpatient substance abuse treatment program clients reporting past 2-year use of OC/OP. A coding guide identified patterns and themes of misidentification in transcribed interviews.

## Results

From December 2010 to January 2014, 57 percent of ASI-MV respondents reporting any OC/OP indicated OC use. In multivariable analyses, OC reporting was greater among Black users (p < 0.05) who were not primarily opioid abusers (p < 0.10), and increased over time among people using opioids as prescribed (p < 0.01). Early post-reformulation, OC use was endorsed by users aged 21–34 and people having recently initiated heroin, but trends reversed over time (p < 0.05). Online forum users reporting NMPU also reported obtaining OC during fall 2013 (18.5%). Qualitative analyses indicated that source of drug identification knowledge, trust and relationship with their drug source, context in which the drug was obtained, and motivations for NMPU contributed to misidentification of OC/OP and other products. “Counterfeits” were noted as a common element of the illicit market and may partially explain endorsement rates, especially early post-reformulation, when street prices for the old formulation (and counterfeits) were high. Cognitive factors such as lacking images (front, back, dosage) and labels, confusion between generics and branded products, and literacy level suggest item-level modifications.

## Conclusions

Possible misclassification of OC/OP and other prescription opioids may be as high as 20 percent among NMPUs and is higher among younger users and limited NMPU experience. Findings have implications for surveillance, policy evaluations, and research using self-reported NMPU. Suggestions include presenting: 1) pill images within a compound in a single view, 2) key milligram increments, 3) street terminology, 4) pill images to scale, both sides, correct coloring, and markings.
